# Faecal microbiota differences between an autochthonous pig breed and a commercial line

**DOI:** 10.1038/s41598-025-13460-y

**Published:** 2025-09-01

**Authors:** Giuseppe Tardiolo, Matteo Daghio, Valentina Riggio, Alessandro Zumbo, Nazareno Antonino Virga, Anna Maria Sutera

**Affiliations:** 1 Department of Veterinary Sciences, University of Messina, Polo Universitario dell’Annunziata, 98168 Messina, Italy; 2 Department of Agriculture, Food, Environment and Forestry, University of Florence, Piazzale delle Cascine 18, 50144 Florence, Italy; 3 The Roslin Institute and Royal (Dick) School of Veterinary Studies, University of Edinburgh, Easter Bush Campus, Midlothian, EH25 9RG UK; 4 Department of Agricultural, Food and Forestry Sciences, University of Palermo, Viale delle Scienze ed. 4, 90128 Palermo, Italy; 5 Meat and Agribusiness Chain Research Consortium, Polo Universitario dell’Annunziata, 98168 Messina, Italy; 6 Department of Chemical, Biological, Pharmaceutical and Environmental Sciences, University of Messina, Viale Ferdinando Stagno D’Alcontres 31, 98166 Messina, Italy

**Keywords:** Nero Siciliano, Crossbred pig, Faecal microbiota, Functional prediction, Inflammatory biomarkers, Biotechnology, Genomics, Metagenomics

## Abstract

The gut microbiota is recognized as one of the most complex microbial ecosystems in mammals. In pigs, the structure of this microbial community significantly influences their overall physiological functions, with breed also contributing to its diversity. This study explores the potential differences in the bacterial composition of the faecal microbiota between two pig breeds: the autochthonous Nero Siciliano (NS) and a commercial crossbreed (CB) (Landrace × Large White). To determine if differences in faecal microbiota composition occur, five NS pigs and seven CB pigs, all managed under identical breeding conditions, were compared at three different time points. The analysis of alpha and beta diversity metrics suggested that time influenced the bacterial profile’s shape. The two pig breeds differed taxonomically in their faecal microbiota composition, showing dynamic changes over time. The prediction of Enzyme Commission numbers was similar between the breeds and time points, but the overall predicted functional profile of the microbial communities differed according to breed and time points. The relative abundances of biosynthesis pathways and macromolecule modification pathways were higher in NS, whereas metabolite precursor and energy generation pathways were higher in CB. Findings of this study can contribute to a better understanding of the faecal microbiota in autochthonous and commercial pigs, providing further insights for breeding management.

**Supplementary Information:**

The online version contains supplementary material available at 10.1038/s41598-025-13460-y.

## Introduction

The study of gut microbiota has received increased attention from the livestock industry and academia, representing one of the most complex microbial communities with a significant impact on the health status of animals^1–3^. These microbial communities play a crucial role in the *One Health* domain by facilitating interactions among humans, animals, and environment, and by participating in co-evolution, co-development, co-metabolism, and co-regulation with their hosts^4^. Because of this interconnectedness, it is important to consider microbiome health in pigs, as well as other livestock species, to improve overall animal health and productivity, which in turn has implications for human health through food safety and zoonotic disease control^4^. It has been reported that the pig gut contains approximately 110 species of microorganisms across 40 families and nine phyla^5^ that interact with the host environment^6^. The complexity and dynamism characterizing the gut microbiota ecosystem are indeed deeply correlated in a symbiotic relationship with the host environment^7–9^. Understanding how microbial species affect host well-being and susceptibility to diseases can offer valuable insights for enhancing animal welfare and optimizing feed utilization within the swine sector^10–13^.

Several investigations have highlighted connections between specific microbial compositions and nutritional outcomes as well as productivity indicators^14–16^. The composition of the gut microbiota is known to affect various aspects of pig health and productivity^17^ including nutrient absorption^13^ growth performance^18^ and immune responses^19^. This relationship underscores the importance of considering genetic factors when studying the gut microbiota and its impact on swine production^20,21^. Notably, the intestinal microbiota processes diverse dietary constituents, yielding nutrients for the host in the form of fermentation by-products and other derivatives, such as amino acids, vitamins, and indole compounds^22,23^.

The composition of gut microbiota in pigs can vary due to age, sex, diet and breeding management, also depending on the gastrointestinal segment considered^5,24^. Several studies indicated that gut microbiota composition can be strictly correlated with host genetics^20,25,26^ but only a few studies have highlighted the possible differences occurring between the bacterial composition of commercial and autochthonous pig breeds^20,25,27^.

The Nero Siciliano (NS) is an autochthonous pig breed, native to the wooded areas of the Nebrodi and Madonie mountains, located on the northern coast of the Mediterranean island of Sicily (Southern Italy)^28^. It is a hardy breed, resistant to diseases and adverse climatic conditions and is highly valued for its high-quality meat production. Unfortunately, as often happens with local breeds, its meat production is either considered a niche production or not enough to address consumers’ demand. So very often this breed gets replaced by more productive breeds and/or commercial crossbreds, that have been selectively bred for optimal productivity traits and extensively studied for their reproduction and production traits, as well as gut microbiota composition^29–31^.

This study aims at exploring the potential variations occurring in faecal microbiota composition between NS pigs and a commercial crossbred (CB) pig (Landrace × Large White), reared under identical breeding management. Comparing the microbiota of NS and CB pigs under identical breeding conditions offers a unique model to disentangle the effects of genetic background on microbial diversity and functionality. Potential similarities and differences in the composition of the faecal microbiota and their functions among the two pig breeds were explored, aiming to identify breed-specific bacteria. Additionally, inflammatory biomarkers such as Haptoglobin (HP), White blood cell (WBC) count and C-Reactive Protein (CRP) obtained from blood samples were correlated with metabolic pathways identified using the functional prediction analysis. By linking microbial profiles to metabolic pathways and inflammatory biomarkers, the study provides a comprehensive analysis of breed-specific microbiota features and their potential implications for pig health and productivity, which could be used for the valorisation of the NS breed.

## Results

To determine the potential differences in the bacterial composition of the faecal microbiota between an autochthonous pig breed (i.e., Nero Siciliano (NS)) and a commercial crossbreed (i.e., Landrace × Large White (CB)), we compiled a dataset consisting of 36 faecal samples from five NS pigs and seven CB pigs, all managed under identical breeding conditions, collected at three different time points (i.e., T0 at the beginning of the study, T1 intermediate point at 30 days, and T2 final point at 60 days). Blood samples collected at the same time points were also available and used to correlate inflammatory biomarkers to metabolic pathways.

### Bioinformatics output, faecal microbiota diversity and taxonomic profile

**Fig. 1:**
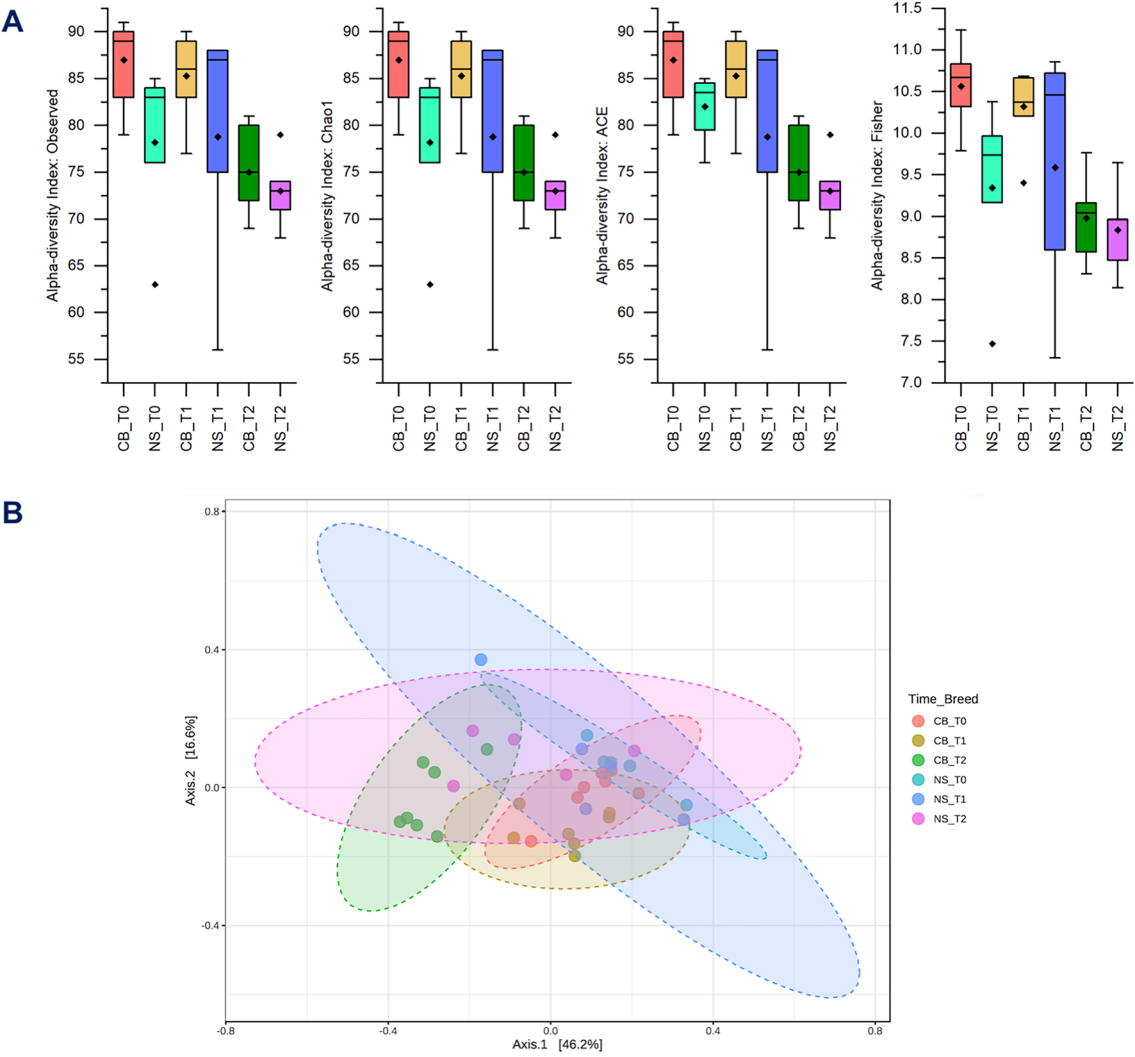
Alpha and beta diversity metrics. (**A**) Alpha diversity indices across the time points (T0, T1, T2) for commercial (CB) and Nero Siciliano (NS) breeds. (**B**) Beta diversity analysis illustrating the differences in microbial community composition between the time points for CB and NS breeds. The axes represent the principal coordinates (Axis 1 and Axis 2) from the PCoA plot, with the percentage of variation explained by each axis indicated in brackets.

The bioinformatics analysis of the metagenomic sequences processed revealed a total of 1,350 amplicon sequence variants (ASVs) classified at the genus level among all samples with counts equal to or greater than 2. The total read count was 1,567,906, with an average of 43,552 reads per sample. The maximum read count per sample was 71,607, while the minimum was 25,375. Regarding the alpha diversity analysis (Fig. 1A), statistical significance was detected when considering the interaction of time points (T0, T1, and T2) and breed. Specifically, the indices that were statistically significant included Observed, Chao1, and Fisher with a *p*-value of 0.003, and the Abundance-based Coverage Estimator (ACE) index with a *p*-value of 0.002. Likewise, the analysis of beta diversity revealed statistical significance when considering the interaction of time points and breed (Fig. 1B). The PCoA plot, based on the Bray-Curtis distance, shows that samples from the two groups of pigs clustered by breed over time, with an overall *p*-value of 0.001 and an R² of 0.49, indicating that 49% of the variance in beta diversity can be explained by the interaction between time points and breed.

**Fig. 2:**
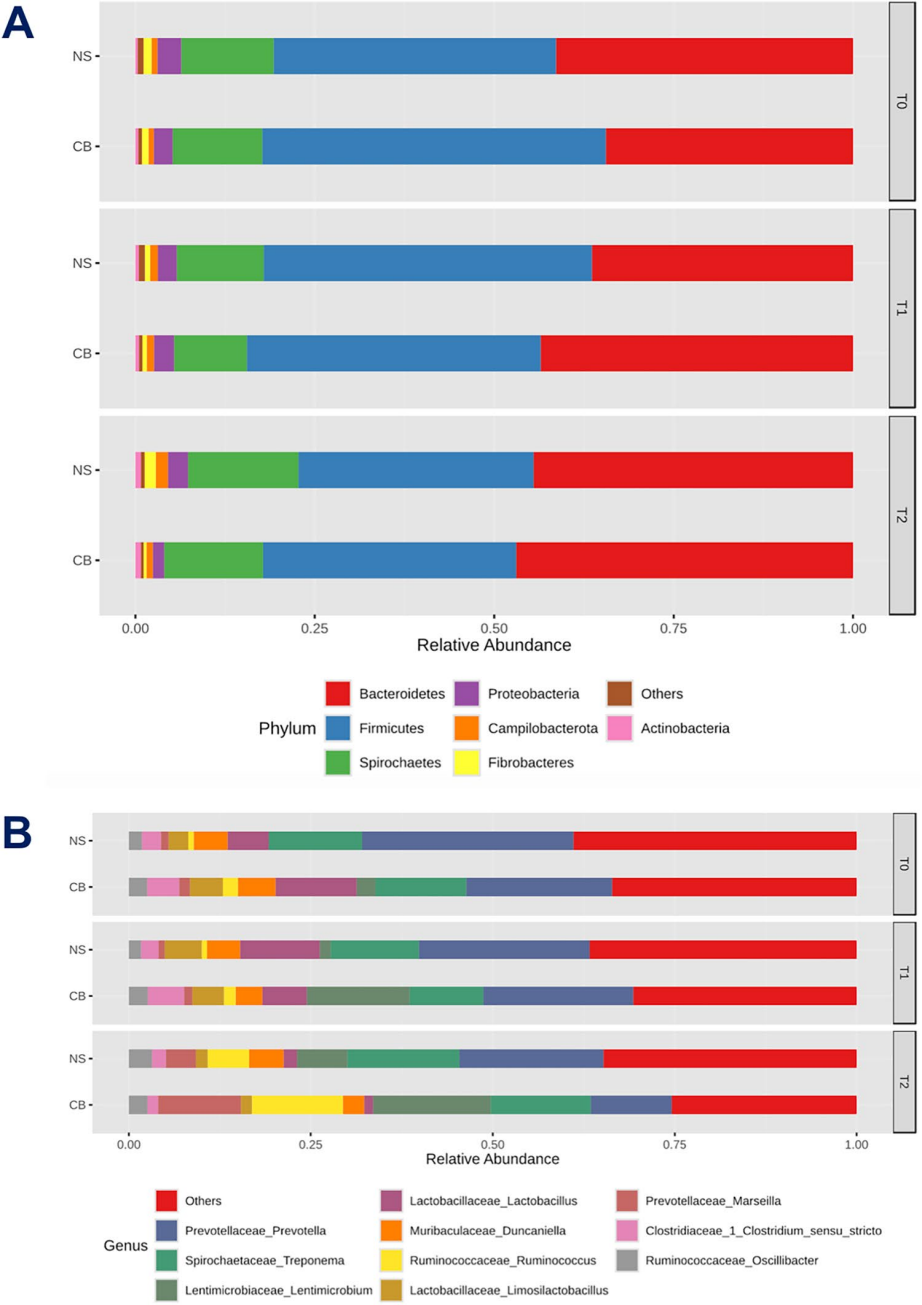
Taxonomic profile. Relative abundances of the most dominant phyla (panel **A**) and genera (panel **B**) in the commercial (CB) and Nero Siciliano (NS) pigs over time. Only top higher taxa are presented. Phyla and genera with lower relative abundances are grouped as ‘Others’.

Figure 2 illustrates the relative abundances of the most abundant bacterial phyla (A) and the top ten genera (B) in the faecal microbiota of CB and NS pigs over time, showing their distribution as a stacked bar chart, providing a clear view of the bacterial composition within each pig breed over time. The overall most abundant phyla in both pig breeds were *Bacteroidetes*, followed by *Firmicutes* and *Spirochaetes*, whereas the most predominant genera were *Prevotella* and *Treponema*. The relative abundances at time points T0, T1 and T2 were 34.4%, 43.5%, and 46.9% for *Bacteroidetes*, 47.8%, 41.0%, and 35.3% for *Firmicutes*, and 12.5%, 10.1%, and 13.7% for *Spirochaetes* in CB pigs. On the other hand, in NS pigs we observed 41.4%, 36.4%, and 44.5% for *Bacteroidetes*, 39.3%, 45.7%, and 32.7% for *Firmicutes*, and 12.8%, 12.1%, and 15.4% for *Spirochaetes*, at T0, T1 and T2, respectively. As for the predominant genera, the relative abundances at time points T0, T1, and T2 were 19.9%, 20.6%, and 11.1% for *Prevotella*, 12.5%, 10.1%, and 13.7% for *Treponema*, and 2.6%, 14.1%, and 16.1% for *Lentimicrobium* in CB pigs. In particular, genera such as *Lactobacillus* (11.2%, 6.1%, and 1.2%), *Ruminococcus* (2.1%, 1.6%, and 12.5%), and *Marseilla* (1.5%, 1.1%, and 11.4%) showed marked fluctuations in their relative abundances over time. Constantly higher relative abundances were found in NS pigs for *Prevotella* (29.1%, 23.4%, and 19.7%) and *Treponema* (12.8%, 12.1%, and 15.4%) at T0, T1 and T2, respectively. However, the presence of the genus *Lentimicrobium* was detected only at T1 and T2 (1.5% and 6.9%, respectively). Compared to CB pigs, in NS pigs only the genus *Lactobacillus* showed a marked fluctuation over time in its relative abundance (5.6%, 10.9%, and 1.8%, respectively).

### Differential abundance analysis

The differential abundance analysis conducted using DESeq2 package to investigate the potential genus-level differences between the two pig genotypes revealed several genera with significant variations. Table S1 details these significant variations in genus abundance, organized by experimental factors, i.e. ‘breed’ and ‘time points and breed’. To determine which bacterial genera increase or decrease, i.e. positively or negatively modulated over time, in NS or CB based on the log2 Fold Change (log2FC) value, both the direction and magnitude of the log2FC must be considered. A negative log2FC value indicates an increase in the bacterial genus in NS relative to CB. Conversely, when comparing NS to CB with time as a covariate, a positive log2FC value indicates an increase in NS.

### Functional prediction analysis of the faecal microbiota

**Fig. 3:**
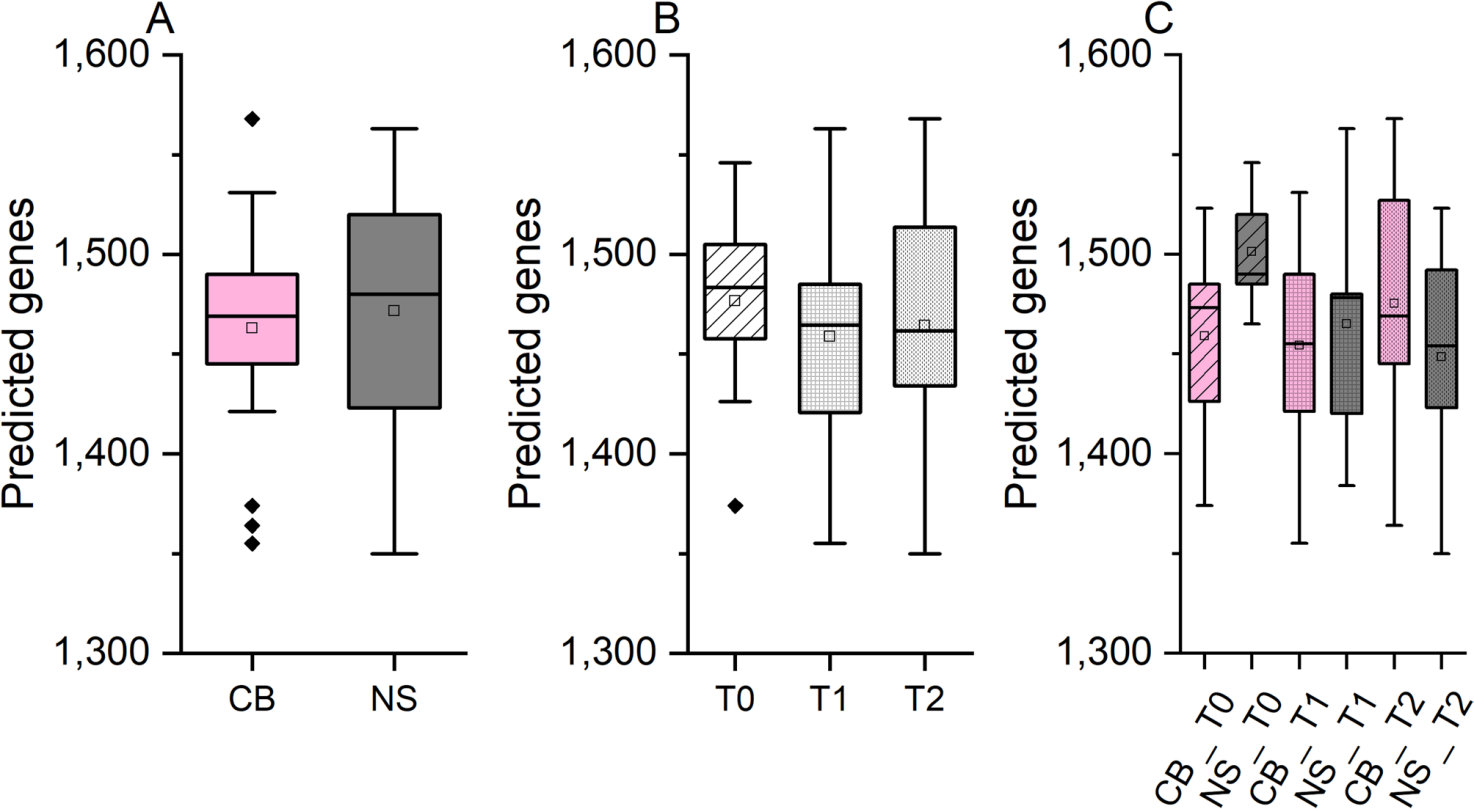
Number of predicted Enzyme Commission (E.C.) numbers in the faecal microbiota. (**A**) For the two pig breeds, (**B**) at different time points, and (**C**) considering the interaction time points and breed. NS, Nero Siciliano; CB, Crossbred; T0, T1 and T2 = different time points, at 0, 30 and 60 days, respectively. The number of predicted E.C. numbers was similar between the breeds and the time points.

**Fig. 4:**
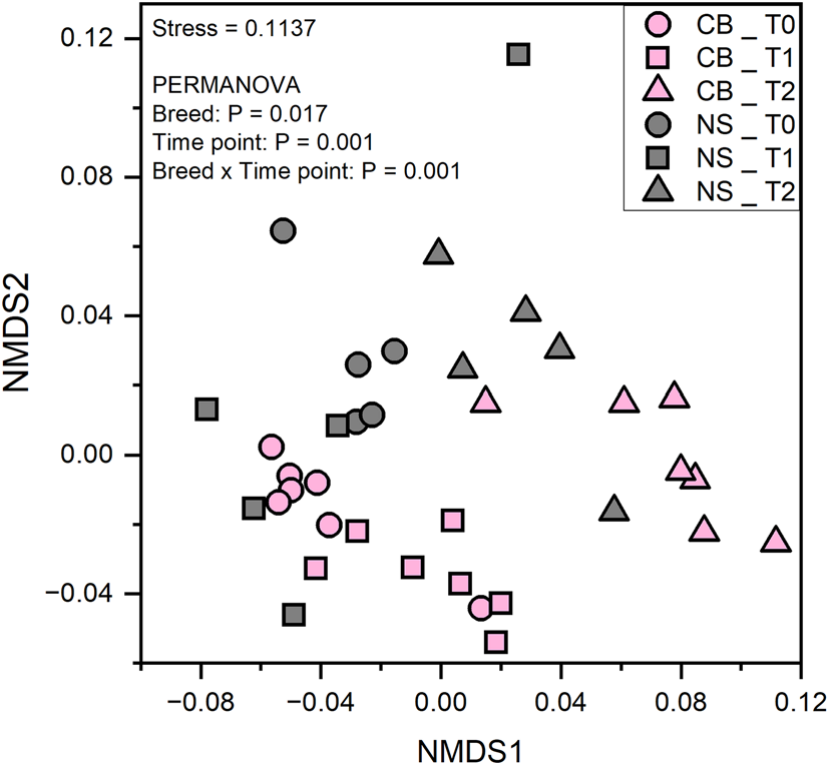
Non-metric multidimensional scaling (NMDS) and a permutational multivariate analysis of variance (PERMANOVA) based on Hellinger transformed E.C. number data. The predicted functional profile of the microbial communities was different according to the breed and to the time points. NS, Nero Siciliano; CB, Crossbred; T0, T1 and T2 = different time points, at 0, 30 and 60 days, respectively.

**Fig. 5:**
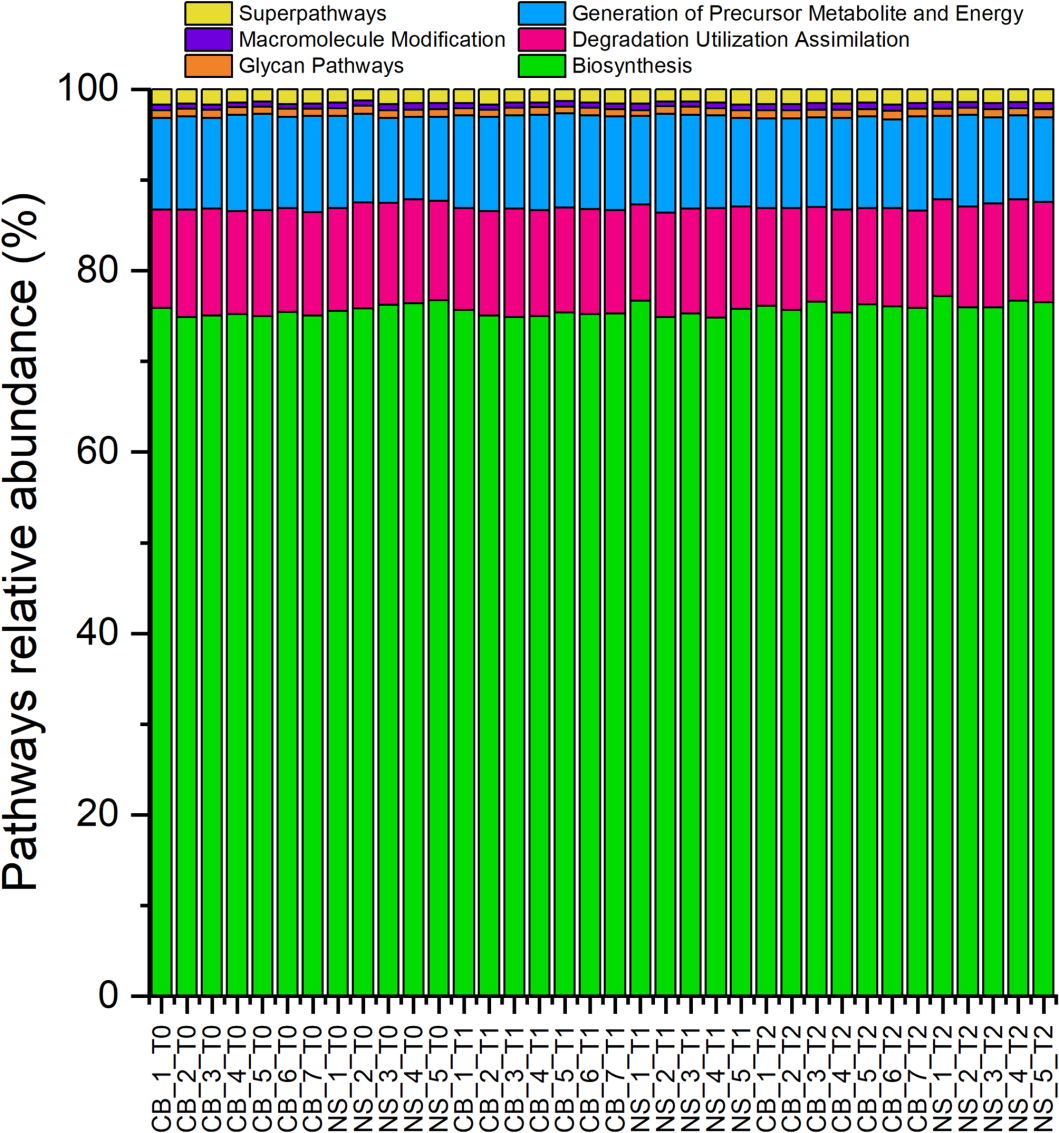
Top classes of the predicted MetaCyc pathways. NS, Nero Siciliano; CB, Crossbred; T0, T1 and T2 = different time points, at 0, 30 and 60 days, respectively.

The number of predicted genes for NS ranged from 1,448 ± 67 (NS_T2) to 1,501 ± 32 (NS_T0), whereas the number of predicted genes for CB ranged from 1,454 ± 56 (CB_T1) to 1,475 ± 66 (CB_T2). No differences were observed in the number of predicted genes (*p* > 0.05) according to breed (Fig. 3A), time point (Fig. 3B) or the interaction breed x time points (Fig. 3C). The analysis of the beta-diversity clearly showed that the predicted metagenomes were different in the two breeds (Fig. 4). Furthermore, a change in the predicted metagenomes was observed also considering the time points and the interaction breed x time points (Fig. 4). The relative abundances of the top classes of the MetaCyc pathways were calculated and reported in Fig. 5, displaying the relative abundances of different metabolic pathways in the gut microbiota of two pig breeds at three different time points. Table S2 reports an overview on the significant pathways encountered. Biosynthesis pathways were the most abundant in all samples (74.81–77.16%) followed by the degradation utilization assimilation pathways (10.42–12.06%) and by the generation of precursor metabolite and energy pathways (9.07–10.87%).

**Fig. 6:**
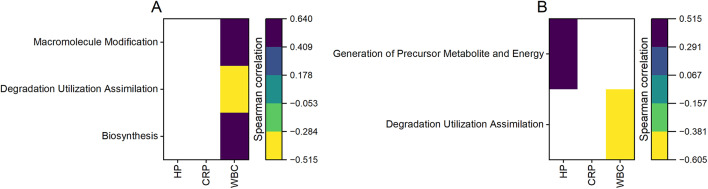
Spearman correlations. Predicted pathways and physiological parameters for the two breeds (CB: **A**; NS: **B**). NS, Nero Siciliano; CB, Crossbred; HP, haptoglobin; WBC, white blood cell count; CRP, C-reactive protein.

The relative abundances of the biosynthesis pathways and of the macromolecule modification pathways were higher in NS, whereas the relative abundances of the generation of precursor metabolite and energy pathways were higher in CB (Table 1). Considering the different time points (Table 2), a higher relative abundance of the biosynthesis pathways and of the macromolecule modification pathways was observed at T2 compared to other sampling points. Conversely, a lower relative abundance of the degradation utilization assimilation pathways and of the generation of precursor metabolite and energy pathways (only compared to T1) was observed in T2. The lowest relative abundance of the degradation utilization assimilation pathways was observed in CB_T2, whereas the lowest relative abundance of the generation of precursor metabolite and energy pathways was observed in NS_T0 and NS_T2 (Table 3). The relative abundance of the biosynthesis pathways and of the macromolecule modification pathways was lower in CB_T0 and CB_T1 comparted to the other groups, with the exception of NS_T1 and NS_T0 (only for the macromolecule modification pathways) (Table 3). The Spearman correlation matrices illustrating the relationships between predicted metabolic pathways and various physiological parameters in the two pig breeds are reported in Fig. 6, with the colour gradient in the heatmap representing the strength and direction of the correlations. The degradation utilization assimilation pathways showed a negative correlation to WBC for the two breeds, whereas the macromolecule modification pathways and the biosynthesis pathways had a positive correlation to WBC in CB (Fig. 6A) and the generation of precursor metabolite and energy pathways had a positive correlation to HP in NS (Fig. 6B).

**Table 1:** Pathways with different relative abundances in the two breeds.

Pathway	CB	NS	*p*-values
	Median (%)	Median (%)	
Biosynthesis	75.37	75.96	0.019
Generation of precursor metabolite and energy	10.27	9.75	0.001
Macromolecule modification	0.58	0.67	0.039

*CB*, Crossbred; *NS*, Nero Siciliano.

**Table 2:** Pathways with different relative abundances at different time points.

Pathway	T0	T1	T2	*p*-values
	Median (%)	Median (%)	Median (%)	
Biosynthesis	75.49^b^	75.22^b^	76.08^a^	0.002
Degradation utilization assimilation	11.44^a^	11.54^a^	10.97^b^	0.001
Generation of precursor metabolite and energy	10.09^ab^	10.33^a^	9.88^b^	0.013
Macromolecule modification	0.59^b^	0.58^b^	0.69^a^	0.001

T0, T1 and T2 = different time points, at 0, 30 and 60 days, respectively. Pathways with different letter (a, b) are significantly different (*p* < 0.05).

**Table 3:** Pathways with a different relative abundance due to the interaction breed x time point.

Pathway	CB_T0	CB_T1	CB_T2	NS_T0	NS_T1	NS_T2	*p*-values
	Median (%)	Median (%)	Median (%)	Median (%)	Median (%)	Median (%)	
Biosynthesis	75.03^c^	75.21^c^	76.03^ab^	76.20^ab^	75.29^bc^	76.49^a^	0.001
Degradation utilization assimilation	11.45^a^	11.55^a^	10.78^b^	11.34^ab^	11.53^ab^	11.10^ab^	0.007
Generation of precursor metabolite and energy	10.27^a^	10.35^a^	9.89^ab^	9.41^b^	10.24^ab^	9.29^b^	0.001
Macromolecule modification	0.55^c^	0.58^c^	0.66^ab^	0.67^abc^	0.61^bc^	0.70^a^	0.001

CB, Crossbred; NS, Nero Siciliano; T0, T1 and T2 = different time points, at 0, 30 and 60 days, respectively. Pathways with different letter (a, b, c) are significantly different (*p* < 0.05).

### Growth performances, and levels of inflammatory biomarkers

**Fig. 7:**
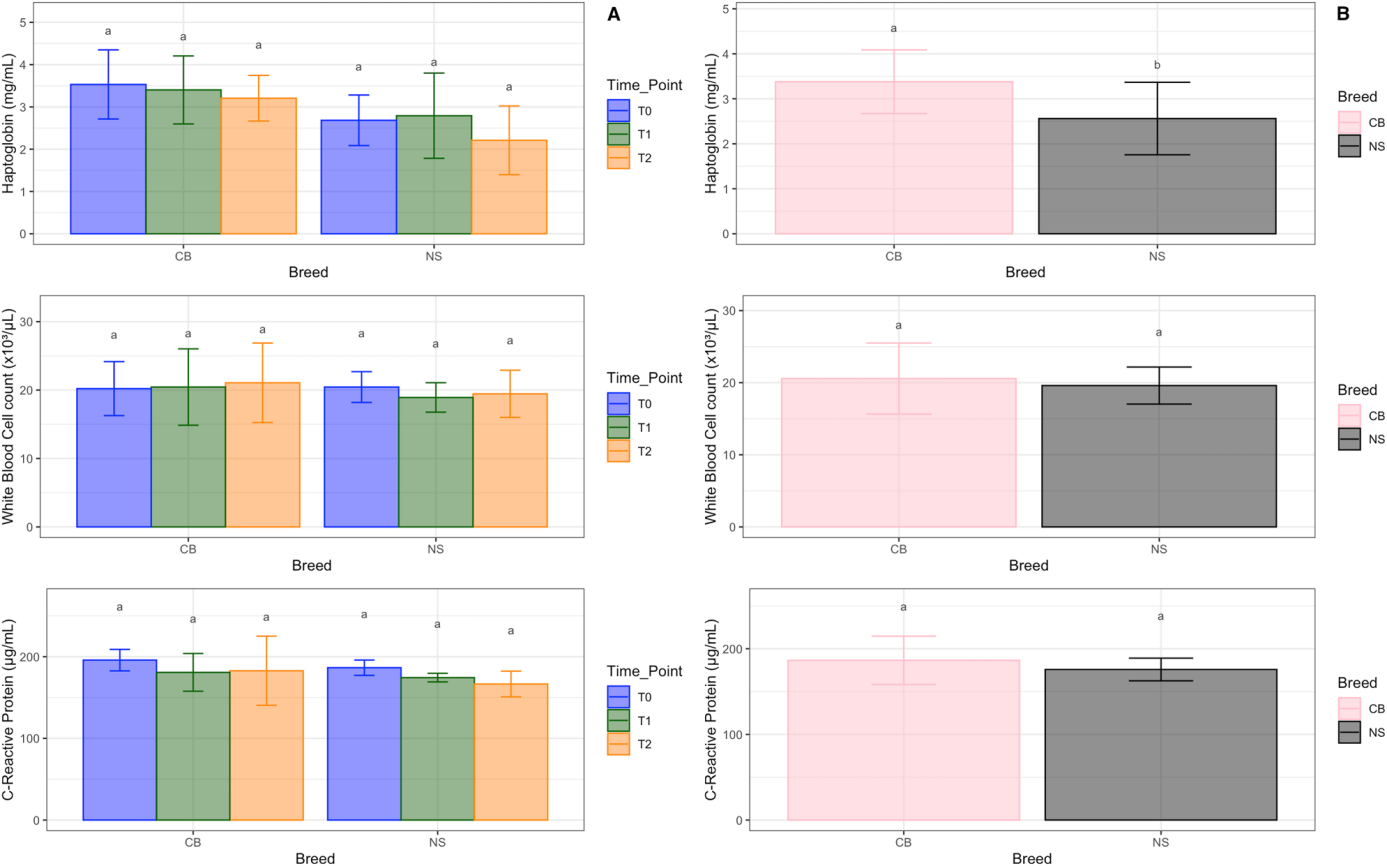
Levels of inflammatory biomarkers Haptoglobin (HP), White Blood Cell (WBC) count, and C-Reactive Protein (CRP) in the two pig breeds. (**A**) Levels of HP, WBC count, and CRP expressed by breed over time, and (**B**) expressed between pig breeds. NS, Nero Siciliano; CB, Crossbred; T0, T1 and T2 = different time points, at 0, 30 and 60 days, respectively. The results are expressed as the mean ± standard error of the mean (*a* and *b* show the statistically significant differences among variables).

The results regarding pig growth performance, such as body weight, average daily gain, and feed conversion rate are those reported for control groups by Tardiolo et al.^32^ for NS pigs, and Sutera et al.^33^ for CB pigs, respectively. The levels of inflammatory biomarkers in the two pig breeds are illustrated in Fig. 7. Figure 7A shows the data for each time point and breed, whereas Fig. 7B compares the overall levels between the two breeds. While no significant differences were observed in the levels of inflammatory biomarkers when considering breeds over time (panel A), HP levels were statistically significant when comparing the two pig breeds (panel B).

## Discussion

The present study explored the variations occurring in the bacterial composition of the faecal microbiota when comparing an autochthonous pig breed (i.e., the Nero Siciliano) and a commercial pig line (Landrace × Large White). The data was collected as part of two previous studies in which we focused on the potential impact of a co-feeding regime using a dairy by-product on the faecal microbiota^32,33^. However, as we aimed at exploring the differences between the two genetic groups, we decided to focus only on the animals identified as control. Liquid whey is a dairy by-product, and therefore not a standardized feed, being affected by several factors, including the cheesemaking management processes^34^. Therefore, any evaluations of the breed effect could be confounded by the supplementation of a by-product that can vary in chemical and microbiological composition. As the pigs used as control were raised under identical management and fed with the same standardized commercial pellet feed^32,33^ we could infer that the potential variation observed in this study, for both the bacterial composition of the faecal microbiota and the abundance profiles of the pathways are due to the breed.

The alpha diversity analysis revealed that only certain indices, i.e. Observed, Chao1, ACE and Fisher, showed statistical significance when considering the interaction of time points and breed, suggesting that the interaction between time and breed impacts specific facets of microbial diversity within samples. Other indices, i.e. Shannon and Simpson indices, that were not statistically significant might measure aspects of diversity that are less sensitive to the specific conditions of breed and time interaction. For example, indices that emphasize evenness more heavily might not show significant changes if the relative proportions of species remain stable despite changes in species richness. The statistical significance of Observed, Chao1, ACE, and Fisher indices suggests that the interaction between breed and time points notably influences species richness and the abundance of both rare and common species, causing dynamic changes in the microbial communities, potentially driven by environmental factors, breed-specific traits, or temporal changes. The NS breed shows greater variability in species richness over time, whereas the CB breed maintains more consistent levels. Studies have shown that host genetics can determine the relative abundance of specific bacterial taxa in the gut microbiome of pigs^35,36^ which supports the observed higher species richness in the NS breed due to its genetic background.

Early-life environmental exposures such as diet, maternal microbiome, and management practices could also have an effect on the gut microbiota^37,38^. However, our pigs were raised under the same management/environment, which should reduce the magnitude of any non-genetic components. The dynamic changes observed in the NS breed over time suggest a more adaptable or fluctuating microbiome, likely responding to dietary or environmental changes. This adaptability is indicative of a microbiome that can adjust to varying conditions, possibly making the animals more resilient against environmental perturbations. Conversely, the stability of the microbiome in the CB breed suggests a more uniform response to environmental factors^39^.

The beta diversity analysis showed distinct clustering, suggesting significant differences in microbial community composition at different time points. In the PCoA plot, the NS breed samples are separated from the CB ones along the primary axis of differentiation (i.e., Axis 1), accounting for most of the variance, namely 46.2% of the total variation. This suggests a significant difference in the overall microbial community structure between the two breeds. For the CB breed, samples at T0 and T1 are closely clustered, while T2 shows some separation, suggesting a shift in microbial community over time. For the NS breed, samples showed more distinct separation, indicating more pronounced temporal changes in microbial composition compared to the CB breed. The distinct clustering of NS and CB breeds suggests inherent genetic or physiological differences that shape their faecal microbiota, that is in agreement with previous findings showing that genetic factors play a crucial role in determining gut microbial composition^35,39^. The more pronounced temporal changes observed in the NS breed suggest a more dynamic microbiome, potentially reflecting greater adaptability to environmental or dietary changes, which also support the results from the alpha diversity analysis. This adaptability could confer advantages in resilience to stressors or dietary modifications^37,40^. Similarly, the relatively stable microbiome composition in the CB breed over time suggests a more consistent microbial community. A stable gut microbiome is crucial for optimal intestinal health and nutrient utilization, as it reduces the risk of disruptions that can lead to disease or poor growth performance^41,42^. Moreover, studies have shown that the gut microbiome of pigs can be influenced by the breed, with certain breeds exhibiting more stable microbial communities under controlled conditions. For instance, variations in the gut microbiota composition among different pig breeds have been linked to differences in feed efficiency and metabolic processes, which are often managed through precise dietary and environmental controls^41,43^.

The taxonomic profile provides insights into the dynamics of faecal microbiota in both pig breeds, highlighting significant changes in the relative abundances of dominant phyla and genera over time. The predominant phyla observed were *Bacteroidetes*, *Firmicutes*, and *Spirochaetes*. This pattern is consistent with findings from other studies, which report *Bacteroidetes* and *Firmicutes* as the most abundant phyla in pigs, playing crucial roles in digestion and metabolic processes^44,45^. Similar microbial compositions were reported for hybrid and specific pathogen-free pigs. Hybrid pigs exhibited substantial changes in gut microbiota composition due to genetic and environmental interactions^46^ with *Bacteroidetes* and *Firmicutes* identified as the dominant phyla, with variations attributed to differences in rearing conditions and genetic backgrounds^45^. These phyla are pivotal in breaking down complex carbohydrates and synthesizing short-chain fatty acids, essential for the host’s energy metabolism and gut health^42,46–48^. Minor phyla showed some fluctuations over time, but these changes were not substantial enough to impact the overall microbial composition significantly. This result is in agreement with other studies indicating that these two phyla, predominant across different conditions and life stages, constitute the majority of the gut microbiota in pigs, playing significant roles in maintaining a balanced microbial ecosystem^42,46^. At genus level, *Prevotella* and *Treponema* were among the most abundant genera. *Prevotella* is highly abundant across all time points in both breeds and it is known for its role in carbohydrate fermentation, whereas *Treponema* is involved in fibre degradation, contributing to the health and productivity of pigs^49^. These genera are commonly reported as dominant in pig microbiota studies, reflecting their essential functions in the gut ecosystem^49^. Temporal changes in microbial composition, particularly the fluctuations in *Lactobacillus*, *Ruminococcus*, and *Marseilla*, highlight the dynamic nature of the bacterial community. Similar temporal shifts have been documented in pigs focusing on age-related microbiota changes, where significant variations were observed across different life stages^44^. In addition, the increased presence of *Prevotella* in NS pigs compared to CB pigs may suggest differences in carbohydrate fermentation capacity and intestinal health, influenced by genetic factors, as these animals were raised under identical farm conditions (i.e., eliminating dietary and environmental variables). Conversely, the low levels of *Lentimicrobium* in NS pigs may suggest different anaerobic intestinal conditions or available substrates, potentially impacting the overall health of the pigs^50^. The genus *Lactobacillus*, present in significant amounts, plays a vital role in maintaining gut health through the production of lactic acid, which inhibits pathogenic bacteria and supports a healthy gut environment^51^. The genus *Treponema* shows notable presence, particularly in the NS breed, suggesting its potential importance in the microbial community of native breeds. Minor genera included *Lentimicrobium*, *Paraprevotella* and *Coprococcus*, present in lower abundances but contributing to the overall diversity and functionality of the faecal microbiota. Other minor genera were *Ruminococcus* and *Oscillibacter*, involved in fibre degradation and short-chain fatty acids (SCFAs) production, which are also essential for gut health^51^. Minor fluctuations are observed in the abundance of genera like *Treponema* and *Lentimicrobium*, particularly in the NS breed, suggesting some temporal variability. The NS breed exhibits higher relative abundances of *Treponema* and *Lentimicrobium* compared to the CB breed, whereas the CB breed shows a more consistent presence of *Lactobacillus* across time points, indicating potential breed-specific factors that promote the stability of this beneficial genus. The presence of key genera like *Prevotella* and *Lactobacillus* across time points in both breeds suggests a resilient gut microbiome capable of maintaining its core functions despite environmental changes. This stability is crucial for digestion, nutrient absorption, and overall gut health^20,21^.

The differential abundance analysis revealed genus-level variations between the two pig breeds (Table S1). The analysis is broken down by experimental factors, including breed-specific and time-point-specific changes. Among the fermentative bacteria, the genera *Saccharofermentans*, *Hungatella*, and *Sporobacter* appear more abundant in NS pigs, highlighting their role in fermentative processes that enhance energy extraction from feed and support gut health^40,52^. Conversely, *Caproiciproducens* and *Alloprevotella*, which are more abundant in CB pigs, contribute to carbohydrate fermentation and SCFAs production^45^. The genera *Roseburia*, *Faecalibacterium*, and *Gemmiger*, all known as butyrate producers, appear more abundant in CB pigs, suggesting that CB pigs might have a gut environment conducive to anti-inflammatory effects and colon health^51^. The presence of butyrate-producing bacteria in higher abundance in CB pigs can potentially lead to improved gut health and resilience against gastrointestinal diseases^53^. *Mucispirillum* and *Streptococcus* genera show varying abundances, with some species potentially contributing to gut health and others being pathogenic. The balance of these genera is crucial for maintaining gut integrity and protecting against infections^54^. *Saccharofermentans* genus, which was significantly more abundant in NS pigs across all three time points (T0, T1, T2), suggests an enhanced ability of their microbiota to ferment complex carbohydrates^52^. Conversely, *Streptococcus* genus was more abundant in CB pigs at T1 and T2, which may indicate a microbiota composition favouring rapid fermentation of simple sugars^54^. The genera *Lentimicrobium* and *Abyssivirga* show a significant increase in NS pigs at early stages, indicating their role in breaking down complex organic matter and aiding nutrient assimilation^55,56^. Conversely, the genera *Macellibacteroides* and *Fournierella* are involved in maintaining a balanced gut microbiota, crucial for the overall digestive health of pigs^57,58^. Finally, the presence of genera like *Adlercreutzia* and *Mediterranea* in higher abundance in CB pigs highlights their roles in maintaining microbial diversity^33,59^.

No significant differences over time were found for the inflammatory biomarkers’ levels for either breed, suggesting that HP levels, WBC counts and CRP levels remained relatively stable across time points for both breeds. The consistency in CRP levels suggests stable acute phase protein levels, reflecting no significant inflammatory response variations over time. On the other hand, a significant difference in HP levels was observed between the CB and NS breeds, thus suggesting breed-specific differences^60^. No significant differences between the two breeds were found for WBC counts and CRP levels. These results are consistent with studies that observed relatively constant levels of acute phase proteins like CRP and HP in control groups of pigs when compared to those exposed to infections or stressors^61,62^ thus indicating that without external stimuli, these markers do not fluctuate significantly. The higher HP levels in CB could indicate a breed-specific predisposition to higher baseline inflammatory states or a response to different environmental stimuli, suggesting that genetic factors can influence acute phase protein levels^60^. A study on Iberian and Large White × Duroc pigs found breed-specific differences in the levels of inflammatory biomarkers and acute phase proteins, suggesting inherent genetic predispositions and variations in immune responses between breeds^63^. The stable CRP levels across breeds and time points suggest that any potential microbiome changes might not significantly impact systemic inflammation as measured by CRP. Findings from other studies indicated that CRP levels can remain stable in the presence of diverse microbiome compositions, highlighting the resilience and stability of this inflammatory marker under different physiological and environmental conditions^42,64^.

In the functional prediction analysis, the number of predicted E.C. numbers did not show significant differences between the two pig breeds, across the time points, or when considering the interaction between breed and time point. Both breeds exhibit similar levels of E.C. diversity, indicating that the functional potential of the faecal microbiome, as predicted by E.C. numbers, is comparable between the two breeds. This suggests that despite genetic and possibly environmental differences between the breeds, their gut microbiomes maintain a similar range of enzymatic functions. Across the time points, the number of predicted E.C. numbers remain consistent. This indicates stability in the functional potential of the faecal microbiome over time, suggesting that temporal factors do not significantly impact the predicted enzymatic diversity in these pig populations. The interaction between breed and time points does not show significant variability in the predicted E.C. numbers. This further supports the finding that the functional diversity of the faecal microbiome is stable and not significantly influenced by either the breed or the specific time points measured in this study. A high number of predicted functions might indicate a high resilience of the microbial community. This resilience is crucial for maintaining essential metabolic and digestive processes within the gut. The ability of the gut microbiome to maintain its functional diversity under various conditions is vital for pig health, as it supports consistent nutrient absorption and metabolic activities^2,65^. A stable gut microbiome ensures that key microbial activities remain unaffected by external changes, thereby supporting long-term health and growth performance in pigs^42,43^. The lack of significant differences between breeds implies that both CB and NS breeds possess a similar number of enzymatic capabilities within their gut microbiomes. The NMDS plot provides a visual representation of the beta-diversity, showing the dissimilarities in the functional profiles of microbial communities between the samples. Each point in the plot represents a sample, and the distance between points reflects the dissimilarity in their predicted E.C. number profiles. The samples from CB and NS breeds and time points form distinct clusters, indicating differences in their microbial functional profiles. The NMDS plot shows clear separation between the samples from the CB and NS breeds. This suggests that the predicted metagenomes, in terms of E.C. numbers, are significantly different between the two breeds. The distinct clustering by breed highlights the influence of genetic factors on the functional potential of the gut microbiota. Within each breed, samples from different time points also show distinct clustering. This indicates that the predicted functional profiles of the microbial communities change over time. The temporal changes suggest dynamic shifts in the microbiome’s functional potential as the pigs mature or in response to environmental factors. The PERMANOVA analysis confirms the statistical significance of the differences observed in the NMDS plot. The results indicate that both breed and time point significantly influence the predicted functional profile of the faecal microbiota. The interaction between breed and time points also shows significant effects, suggesting that the changes in functional potential are not uniform across breeds and time points. Across all samples, certain pathways are consistently predominant, indicating core metabolic functions essential for gut microbiome activity. These pathways likely include carbohydrate metabolism, amino acid metabolism, and energy production pathways. The dominant pathways suggest essential microbial functions necessary for maintaining gut health and overall metabolic homeostasis. The relative abundance of specific pathways varies between CB and NS breeds. This variation reflects the influence of genetic factors on the functional potential of the gut microbiome. Within each breed, the relative abundance of pathways changes over time. These temporal dynamics indicate adaptive shifts in the microbiome’s functional capabilities in response to dietary changes, growth stages, or environmental factors. The interaction between breed and time point reveals how the combination of genetic background and temporal factors influences the functional profile of the microbiome. Significant interactions suggest that the pathways’ relative abundances are not solely dependent on breed or time point but are affected by the combined influence of both. The differences in pathway composition between CB and NS breeds suggest that each breed’s microbiome is tailored to support distinct metabolic functions. This breed-specific functional potential could be leveraged to enhance productivity and health through tailored management practices^2^. The results demonstrate that both the composition and functional profiles of the gut microbiota are significantly influenced by breed, time points, and their interaction. This underscores the importance of considering both genetic and temporal factors when studying the gut microbiome in pigs. The significant differences between the CB and NS breeds indicate that genetic factors play a crucial role in shaping the functional potential of their microbiome. This finding can be leveraged to develop breed-specific dietary and farm management practices to optimize gut health and productivity.

We have found that HP positively correlates with macromolecule modification and energy generation but negatively with degradation and assimilation. WBC count and CRP show varied correlations, indicating distinct roles of microbial functions in inflammation. These findings suggest that specific microbial activities are closely linked to the host’s inflammatory status, reflecting the complex interaction between gut microbiota and host physiology. Further investigation into these relationships could provide insights into the mechanisms underlying microbial influence on inflammation and health.

The significant changes in microbiota composition and function over time highlight the dynamic nature of the gut microbiome. These temporal shifts are essential for understanding how the microbiome adapts to developmental changes, dietary modifications, and environmental factors. The significant interaction between breed and time points suggests that the microbiome’s response to temporal changes is breed-dependent. This highlights the need for tailored interventions that consider both the breed and specific developmental stages to effectively manage gut health. The differences in correlation patterns between the two breeds underscore the genetic and metabolic distinctions that influence their physiological responses. These breed-specific correlations can guide targeted nutritional and management interventions to optimize health and productivity in each breed.

## Methods

### Ethics declarations

All experimental protocols were approved by the Animal Experiment Ethics Committee of the University of Messina (authorization number 055_2021; 6 May 2021). All methods were carried out in accordance with relevant guidelines and regulations, specifically following the European Directive 2010/63/EU for the protection of animals used for scientific purposes. Additionally, the study adheres to the ARRIVE guidelines (https://arriveguidelines.org) for reporting animal research. The experimental procedures, including animal management and sample collection, were conducted with strict adherence to these ethical standards.

### Experimental design and sample collection

All individuals were homogeneous for sex (female), body weight (average initial body weight: 19.4 ± 1.92 kg for NS pigs and 20 ± 1.5 kg for CB pigs), and age (58 ± 2 days for NS pigs and 60 ± 2 days for CB pigs). Both groups were raised in the same farm under identical environmental and management conditions and were fed the same commercial pellet feed. The study lasted 60 days. Faecal and blood samples from five NS pigs and seven CB pigs (Landrace × Large White) were collected at three time points (T0 at the beginning of the study, T1 intermediate point at 30 days, and T2 final point at 60 days), resulting in a total of 36 samples. These samples served as control groups in two previous studies examining the effect of a co-fed diet on faecal microbiota composition (i.e., Tardiolo et al.^32^ for NS pigs, and Sutera et al.^33^ for CB pigs). More details about the farm and breeding management, experimental design, feeding regime (for the control group), faecal sample collection, and growth performance can be found in Tardiolo et al.^32^ and Sutera et al.^33^. Blood sample collection and laboratory procedures for inflammatory biomarkers evaluation (i.e., HP, WBC count, and CRP) were as described by D’Alessandro et al.^66^.

### Bioinformatics and statistical analysis

The bacterial V3-V4 hypervariable regions of the 16 S rRNA gene were sequenced using Illumina’s MiSeq v3 platform in 2 × 300 bp paired-end mode (San Diego, CA, USA), as described by Tardiolo et al.^32^ and Sutera et al.^33^. The raw metagenomic sequencing data have been deposited in the international Sequence Reads Archive database under the study accession numbers SUB12344409 (BioProject ID PRJNA909983, Tardiolo et al.^32^) and SUB12998317 (BioProject ID PRJNA951774, Sutera et al.^33^), respectively. After downloading the raw data from the pigs’ control groups, the Illumina paired-end sequences were denoised, dereplicated, and quality-filtered for chimeras before being assembled into error-corrected amplicon sequence variants (ASVs) using the high-resolution Divisive Amplicon Denoising Algorithm 2 (DADA2) package (v1.26)^67^ in RStudio. For downstream analysis, only reads meeting the criteria of a quality score of 20 or above (Q ≥ 20) and a minimum length of 50 base pairs (≥ 50 bp) were included. Subsequently, the assembled ASVs were taxonomically assigned from the phylum to genus level using the Ribosomal Database Project for the Bacterial 16 S rRNA gene^68,69^.

Statistical analyses and data visualization were conducted in RStudio v4.4.1^70^. Phyloseq^71^ and ggplot2^72^ R packages were used to estimate and plot the diversity metrics across all samples. Differences in the alpha diversity metrics were estimated using the Kruskal-Wallis test^73^; whereas beta diversity was calculated using a principal coordinate analysis (PCoA) as the ordination method based on the Bray-Curtis distance^74^ applying a permutational multivariate analysis of variance (PERMANOVA). The features for relative abundances at various taxonomic levels across samples were filtered based on their abundance (minimum count of 4) and prevalence (20% prevalence across all samples). The differential abundance analysis was performed using the DESeq2 package^75^ to identify significant differences by correlating the experimental factors for breed and time points and breed. Despite the possibly high false positives rate DESeq2 is considered an appropriate method when the sample size is low (i.e., lower than 8) because of the high power^76^. In the analysis of the levels of the inflammatory biomarkers, the collected data for HP, WBC count, and CRP underwent to statistical examination and were presented as the mean values of three replicates for each variable, along with their standard errors. A two-way analysis of variance (ANOVA) was employed to test hypotheses at a 1% significance level. Following this, Tukey’s HSD test was used post hoc to compare the means between the breeds over different time points concerning the dependent variables.

### Functional prediction

PICRUSt2 software^77^ was used for the functional prediction analysis with default parameters. The ASVs sequences and abundances were used for the prediction of the Enzyme Commission (E.C.) number metagenome and the E.C. number were used to calculate the MetaCyc pathways abundance. Statistical elaboration was performed using the vegan package, v2.6.4^78^. A non-metric multidimensional scaling (NMDS) and a PERMANOVA based on Hellinger transformed E.C. number data were performed using the metaMDS and the adonis2 functions, respectively. Furthermore, an ANOVA was performed to detect differences in the number of predicted genes between samples groups (i.e., breed, time point and interaction of breed x time point). The relative abundances of the top classes of the MetaCyc pathways were calculated and the pathways with different relative abundances between the conditions (i.e., breed, time points and interaction of breed x time points) were identified by a Kruskal-Wallis test^73^ and by a post-hoc Dunn test with the Benjamini-Hochberg correction for multiple comparison^79,80^. In addition, the Spearman correlations were performed to identify pathways correlated to physiological data (i.e., HP, WBC count and CRP). All statistical analyses considered *p*-values of ≤ 0.05 as statistically significant.

## Conclusions

This study explored the potential breed-specific differences in the faecal microbiota of an autochthonous pig breed (NS) and a commercial line (CB) over time. Our findings suggest that the NS faecal microbiota is characterized by greater variability, potentially reflecting its adaptation to extensive rearing systems, while the CB pigs exhibit a more stable faecal microbiota under uniform conditions. These differences may potentially influence traits of economic relevance, such as meat quality and metabolic efficiency. The potential breed-specific differences observed underscore the importance of considering genetic background in microbiome-related health management practices, enabling us in developing future strategies to enhance gut health through targeted dietary interventions and management practices in swine production.

## Supplementary Information

Below is the link to the electronic supplementary material.

Supplementary Material 1

Supplementary Material 2

## Data Availability

Findings of the present study are available from the corresponding author upon reasonable request. Raw metagenomic sequences used in this study were downloaded by the Sequence Reads Archive database for NS pigs by Tardiolo et al.^32^ 10.3390/ani13040642, and for CB pigs by Sutera et al.^33^ 10.3390/ani13111750.
