# Supplementary material for: Faecal microbiota differences between an autochthonous pig breed and a commercial line
Source: Sci Rep. 2025 Sep 1;15:32176. doi: 10.1038/s41598-025-13460-y (PMC12402455; doi:10.1038/s41598-025-13460-y)
Supplement: Supplementary file 2 — Supplementary Material 2 [file 41598_2025_13460_MOESM2_ESM.pdf]

Table S1. Overview of the differential abundance analysis reporting the significant variations at genus-level.

Genus	Log2 Fold Change*	*p*-values	FDR
	Breed		
	NS vs CB		
Saccharofermentans	7.53	1.36×10^{-5}	1.03×10^{-3}
Streptococcus	1.93	1.95×10^{-5}	1.03×10^{-3}
Caproiciproducens	-4.15	2.98×10^{-5}	1.05×10^{-3}
Hungatella	6.46	2.31×10^{-4}	5.03×10^{-3}
Sporobacter	1.48	2.37×10^{-4}	5.03×10^{-3}
Alloprevotella	-1.11	2.18×10^{-3}	3.86×10^{-2}
	Time points and Breed		
	NS_T0 vs CB_T0		
Lentimicrobium	27.59	2.88×10^{-34}	3.1×10^{-32}
Abyssivirga	23.09	2.95×10^{-17}	1.56×10^{-15}
Hungatella	21.28	1.84×10^{-14}	6.52×10^{-13}
Macellibacteroides	9.12	1.64×10^{-9}	4.36×10^{-8}
Streptococcus	3.82	5.72×10^{-9}	1.21×10^{-7}
	NS_T1 vs CB_T1		
Saccharofermentans	23.65	1.19×10^{-14}	1.27×10^{-12}
Streptococcus	2.18	7.71×10^{-4}	4.1×10^{-2}
	NS_T2 vs CB_T2		
Mucispirillum	-22.09	6.20×10^{-16}	6.57×10^{-14}
Dialister	-21.93	1.37×10^{-10}	7.29×10^{-9}
Roseburia	-6.84	3.25×10^{-7}	1.15×10^{-5}
Blautia	-4.84	1.24×10^{-5}	3.29×10^{-4}
Gemmiger	-2.01	1.68×10^{-4}	3.57×10^{-3}
Faecalibacterium	-2.74	2.46×10^{-4}	4.35×10^{-3}
Caproiciproducens	-5.97	3.39×10^{-4}	5.14×10^{-3}
Mediterranea	-6.94	4.52×10^{-4}	5.98×10^{-3}
Fournierella	-2.74	6.46×10^{-4}	7.61×10^{-3}
Sporobacter	1.99	4.06×10^{-3}	4.08×10^{-2}
Adlercreutzia	3.05	4.23×10^{-3}	4.08×10^{-2}

*Changes in genus-level abundance sorted by experimental factors; NS = Nero Siciliano; CB = Crossbred; T0, T1 and T2 = different time points, at 0, 30 and 60 days, respectively; FDR = False Discovery Rate; only genera that are significant for both *p*-values and FDR are reported.

Table S2. Detailed list of the significant pathways identified considering the following experimental factors: breed, and breed and time point. It also includes the relative abundances of each pathway and the results of the Spearman correlation.
